# Person-based co-design of a decision aid template for people with a genetic predisposition to cancer

**DOI:** 10.3389/fdgth.2022.1039701

**Published:** 2022-11-23

**Authors:** Kate Morton, Kelly Kohut, Lesley Turner, Sian Smith, Emma J. Crosbie, Neil Ryan, Chloe Grimmett, Diana M. Eccles, Claire Foster, The CanGene CanVar Patient Reference Panel, The International Lynch Decision Aid Stakeholder (LDAS) Panel

**Affiliations:** ^1^ Centre for Psychosocial Research in Cancer, Health Sciences, University of Southampton, Southampton, United Kingdom; ^2^ Patient and Public Contributor, Southampton, United Kingdom; ^3^ Aston University, College of Health and Life Sciences, School of Optometry, Birmingham, United Kingdom; ^4^ Department of Gynaecology, St Mary's Hospital, Manchester University NHS Foundation Trust, Manchester Academic Health Science Centre, Manchester, United Kingdom; ^5^ Division of Cancer Science, Faculty of Biology, Medicine and Health, University of Manchester, Oxford Road, Manchester, United Kingdom; ^6^ College of Medicine and Veterinary Medicine, University of Edinburgh, United Kingdom; ^7^ Department of Medicine, University of Southampton, Southampton, United Kingdom

**Keywords:** decision aid (DA), template, genetic predisposition, cancer, patient and public involvement (PPI), person-based approach

## Abstract

**Background:**

People with genetic predispositions to cancer are faced with complex health decisions about managing their risk. Decision aids can support informed, values-based decisions, alongside shared decision-making with a clinician. Whilst diagnoses of genetic predispositions to cancer are increasing, there is no scalable decision aid to support these people. This paper presents an accessible, relevant decision aid template which can be adapted for different predispositions to cancer.

**Methods:**

The decision aid template was co-developed with 12 patients affected by cancer and informed by empirical and theoretical literature. In addition, consultations were conducted with a further 19 people with Lynch syndrome; a specific genetic predisposition to cancer. Clinical stakeholders were consulted regularly. Coulter's framework for decision aid development guided the process, and these activities were complemented by the International Patient Decision Aid Standards, and the latest evidence on communicating risk in decision aids. Programme theory was developed to hypothesise how the decision aid would support decision-making and contextual factors which could influence the process. Guiding principles co-developed with the patient panel described how the decision aid could effectively engage people.

**Results:**

The in-depth co-design process led to the identification of five core components of an accessible decision aid template for people with a genetic predisposition to cancer: defining the decision; a table showing implications of each option; optional further details such as icon arrays to show tailored risk and personal narratives; values clarification activity; and a summary to facilitate discussion with a clinician. Specific guidance was produced describing how to develop each component. The guiding principles identified that the decision aid template needed to promote trust, reduce distress, and be comprehensive, personally relevant and accessible in order to engage people.

**Conclusion:**

Adopting a co-design process helped ensure that the decision aid components were relevant and accessible to the target population. The template could have widespread application through being adapted for different genetic predispositions. The exact content should be co-designed with people from diverse backgrounds with lived experience of the specific predisposition to ensure it is as useful, engaging and relevant as possible.

## Introduction

As genetic testing becomes increasingly available and prioritised in mainstream healthcare (1–4) more people are being diagnosed with an increased genetic risk of developing cancer (5). Once diagnosed, their at-risk relatives become eligible for genetic testing too. People with a genetic predisposition to cancer can be faced with an array of complex decisions about their health, including whether and when to have risk-reducing surgery, whether to take medication to reduce risk, and how best to engage in available screening (6–8). Decision aids could be used alongside consultation with healthcare professionals to support these people to make informed, values-based decisions about their options (9). Decision aids have been shown to reduce decisional conflict, improve knowledge and facilitate more realistic expectations about healthcare (9).

However, a systematic literature review identified only six decision aids to support people living with a genetic predisposition to cancer, all of which targeted BRCA carriers (10), demonstrating the need for improved decision support resources for people with other genetic predispositions to cancer. Within the field of genetics, the identification of new variants and rapidly evolving evidence is common (11), suggesting that the development of a decision aid template grounded in users’ preferences and support needs which could be rapidly adapted for different variants could have widespread application. This template would provide a set of core components for the decision aid, as well as guidance about how to write the content. Decision aid templates are also a cost-effective solution given the intensive process of developing new decision aids from scratch (12).

The International Patient Decision Aid Standards (IPDAS) provide guidance regarding best practice for developing decision aid components (13). The IPDAS state that decision aids should be based on evidence, developed using a systematic process, written in plain language, and include information about options, probabilities of outcomes, and values clarification. The Ottawa Patient Decision Aid offers a generic template which meets the IPDAS criteria and allows the decision aid developer to “fill in the gaps” (14). However, this template does not attend to important nuances of *how* components are worded or presented to ensure acceptability to the target population. Furthermore, the same components may not be appropriate for all decision aids as health decisions and the context in which they are made vary enormously (15–17). There is a need for a template specific enough to ensure clear consistency in the decision aid components, but flexible enough to allow content to be tailored for the specific genetic predisposition.

The template planning and development process was informed by several intervention development approaches. We used tools from the Person-Based Approach, which promotes iterative engagement with the target population to develop in-depth understanding of their beliefs about the health condition and related behaviours, and ensure that the intervention is as engaging and meaningful as possible (18). We also followed Coulter's framework for decision aid development (12) and referred to the updated Medical Research Council (MRC) framework for developing and evaluating complex interventions to ensure that methodological and theoretical considerations were incorporated, such as stakeholder engagement, refining the decision aid template and using programme theory to show the anticipated mechanisms through which the template may support informed decision-making (19).

This paper describes a co-development process to identify the core components and guiding principles for a decision aid template for people living with a genetic predisposition to cancer.

## Materials and method

### Design

The iterative development of the decision aid template took place from February 2021 to July 2022 and involved multiple cycles of optimisations based on feedback from the target user population and clinical stakeholders. This paper reports the development process using the DEVELOPTOOLS reporting checklist, which specifically focuses on the design of decision aids (20).

### Co-development contributors

#### Patient panel members

Throughout the decision aid development, we worked closely with a patient panel of 12 patients affected by cancer and with an interest in genetics. Some members of our patient panel were invited directly by our panel chair due to a known interest in cancer genetics from previous research projects, some expressed interest to the researchers as they wanted to share their perspective on living with a genetic predisposition, whilst others responded to adverts from patient-led charities such as Lynch Syndrome UK, the National Cancer Research Institute Consumer Forum, and Independent Cancer Patients' Voice who shared a summary of the research *via* social media or mailouts.

Of the 12 people, four were male, ages ranged from early-20s to mid-50s, eight had a genetic predisposition to cancer, and seven had had cancer.

#### Public involvement contributors

In order to engage with a wider group from our target population, we organised online discussion groups with 19 people with Lynch syndrome; a specific genetic predisposition to cancer. Lynch syndrome is caused by a pathogenic variant in one of five genes, causing increased risks for several cancers including endometrial, ovarian, and colorectal (21). Thirteen of the 19 public involvement contributors with Lynch syndrome completed an optional demographics form after the discussion. This showed a varied distribution in terms of gender and clinical demographics, but most were white and aged between 41 and 60 years, see Table 1.

**Table 1:** Demographics and clinical information for the public involvement contributors (*n* = 13).

Demographic factor	Distribution (Total *n* = 13)
Age	26–40 years: 3
	41–60 years: 9
	61–70 years: 1
Gender	Female: 8
	Male: 5
Ethnicity	White: 12
	Indian: 1
Education	Before finishing school: 1
	After finishing school: 10
	Post-graduate studies: 2
Context of genetic predisposition diagnosis	Predictive testing due to family member diagnosed: 8
	Diagnostic testing following a cancer diagnosis: 5
Experience of cancer	Colorectal cancer: 6
	Endometrial cancer: 1
	No cancer: 6

#### Clinical/decision aid stakeholders

A world-leading clinician in cancer genetics formed a core part of our research team and inputted to all decisions about the decision aid (DE). In addition, a panel of four stakeholders were recruited to input to the decision aid template development, including gynaecologists with expertise in risk-reducing surgery (EC, NR), a health statistician (PM), and a health psychologist with expertise in developing low literacy decision aids (SS). Stakeholders were invited based on our research team's connections and knowledge of experts in the field.

### Co-development procedures

Having risk-reducing surgery to remove the womb and ovaries after diagnosis with Lynch syndrome was chosen as the example decision to inform the decision aid template development. This was chosen because:

a. Diagnosing Lynch syndrome has been identified as a priority by NHS England, as it is one of the most common hereditary cancer predispositions and most people with Lynch syndrome have not been diagnosed (22)
b. Few resources exist for people with Lynch syndrome (10)
c. Whether to have risk-reducing surgery is often a decision faced by people with a genetic predisposition to cancer

Planning and developing the decision aid template was an iterative process which followed Coulter's framework for decision aid design (12). This is described in Table 2 with reference to how theory, evidence, and public and stakeholder involvement was incorporated throughout. These steps often occurred in parallel.

**Table 2:** Co-development process based on coulter's framework for decision aid development.

Coulter's Framework for Decision Aid Development (12)	Methods
Scoping	The specific decision was defined based on the latest guidance for people with Lynch syndrome, informed by the clinical stakeholders specialising in Lynch syndrome management (EC, NR).
	The need for support in making this decision was recognised by the public involvement contributors with Lynch syndrome.
	A behavioural analysis of the six decision aids identified for people with a genetic predisposition to cancer *via* a systematic review (10) confirmed that none of these met all the IPDAS, supporting the need for a template for this population.
Steering Group	A patient panel including 12 patient contributors, and a stakeholder group including clinicians and low literacy decision aid experts were set up at the project outset (see Section 2.2)
Design 1 and 2: Assess Decisional Needs	Consultations were conducted with 19 people with Lynch syndrome *via* online discussion groups or telephone, co-facilitated by members of the patient panel. Discussions explored perceptions about managing cancer risk, and feedback on components from existing patient support resources* to identify the type of support people wanted.
	Discussions with the patient panel about how the decision aid could best engage its target audience informed the development of guiding principles [an intervention planning tool from the Person-Based Approach to promote engagement with interventions (23)].
	Relevant literature was signposted by clinician and decision aid stakeholders.
Design 3: Determine Format and Distribution Plan	Examples of existing decision aids* were reviewed with the patient panel to identify which components were useful, and important considerations in how these should be designed.
	Planned decision aid components were mapped back to the IPDAS and Ottawa Decision Support Framework to ensure key components had not been omitted.
	A programme theory was developed to show how the decision aid was hypothesised to improve decisional outcomes (19).
	Ongoing discussions with clinical stakeholders and patient panel about how these resources can best be implemented in mainstream care.
Design 4: Review and Synthesise Evidence	Guidance from clinical stakeholders regarding the latest evidence and guidelines around healthcare options.
Prototype Development	Detailed small group discussions about three iterative versions of decision aid with patient panel.
	Detailed written feedback from the patient panel on each version of the decision aid was also incorporated.
	Detailed written feedback on the decision aid content from clinical and decision aid literacy stakeholders (EC, NR, SS, PM).
	All feedback was collated in a table of changes (tool from the person-based approach) to help identify where changes were needed to improve the accessibility, relevance and usefulness of the decision aid.
Alpha Testing	Alpha and beta testing will be undertaken with clinicians and people from the target population, including think-aloud interviews, but is not reported in this paper.
Beta Testing	

* The existing patient support resources were selected to provide examples of a range of the core components of decision aids, defined as: “At a minimum decision aids describe the health condition or problem; make explicit the decision; provide information on options, benefits, and harms; and help patients clarify which benefits and harms matter most. Optional features in decision aids are probabilities of outcomes of options, narratives describing patients’ experiences with making decisions, and guidance in the process of decision making” (24).

In order to explore the transferability of the new template, it was then used to develop a decision aid for people considering whether to take aspirin to help manage their genetic predisposition to cancer. Using the core components of the decision aid identified for risk-reducing surgery, we followed a similarly iterative process to plan and optimise the decision aid content, working closely with our patient panel, leading clinical stakeholders specialising in the use of aspirin for managing colorectal cancer risk (JB, KMo, and DC), and a health researcher exploring patients’ and clinicians' perspectives of aspirin for people with Lynch syndrome (KL).

Supplementary material A includes more detail about the engagement methods with the stakeholders.

### Process for incorporating public and stakeholder perspectives

All evidence and feedback from the patient panel, public involvement contributors, and stakeholders was captured in an intervention planning table during the planning phase, and a table of changes during the development phase. This enabled transparent, rigorous recording of the co-development process, and acted as a record of the changes made in each iteration of the decision aid and the rationale behind them (23).

## Results

### Decision aid template core components

The co-design process identified five components of a decision aid template for supporting people with a genetic predisposition to cancer (©University of Southampton). These were used to develop a decision aid for risk-reducing surgery, and successfully adapted to develop a decision aid for taking aspirin with the same components.

The components are described below with a definition, rationale and example for each. Table 3 shows the sources of evidence informing the inclusion of each component.

**Table 3:** Sources of evidence informing the inclusion of each component of the decision aid template (©University of Southampton).

	Patient panel	Public involvement consultation	Clinical stakeholders	Evidence	Theory	IPDAS
1. Defining the decision (including the option to do nothing)	x			x		x
2. Table presented at the outset to show the implications of each option, using merged boxes where information is the same	x	x	x	x	x	x
3. Optional further details such as tailored risk, symptoms, and personal stories	x	x	x	x	x	x
4. Values clarification activity, including tailored feedback	x	x		x	x	x
5. Tailored summary to facilitate discussion with a healthcare professional	x		x	x		x

Boxes 1-5 show example content from each component of the prototype decision aid. The content is still being revised in line with feedback from the target population.

1. Defining the decision (including the option to do nothing)

Definition: A clear explanation that there is a decision to be made, which includes the option of doing nothing, and that this decision aid can help you to think about your options.

Rationale: The patient panel indicated that it was important to set the context by letting people know there was a decision to be made, before providing any information. This was seen as particularly relevant for people newly diagnosed with a genetic predisposition, who needed to understand that something about their health has changed and there are now various options available.

This suggestion is consistent with the step in the Ottawa Decision Support Framework of clarifying the decision and inviting participation (14), and the IPDAS criterion of explicitly stating the decision that needs to be considered (13). It was further supported by recommendations from qualitative research exploring perceptions of a decision aid for bowel cancer screening, which suggested that people may be unsure about the purpose of a decision aid and therefore clearly explaining the decision itself at the outset is important (24). Further, in line with IPDAS, the decision aid needed to include the option of doing nothing (13, 25).

Therefore, the decision aid template began by explaining the decision, and how this decision aid can support you (See Box 1).

Box 1 **Example of defining the decision.**

What are my options?

• People with Lynch syndrome can choose to have an operation to remove their womb (the organ where a baby grows. This is also sometimes called the uterus) and ovaries (the glands that produce eggs and some hormones).
• You can choose to:
  ○ Have the operation at a time that is right for you.
  ○ Not have the operation
  ○ Or wait to decide later when you are ready.
• This decision is personal. There is no right or wrong decision as everyone is different.
• This session will help you decide what is right for you at the moment.

(2) A table presented at the outset to show the implications of each option, using merged boxes where information is the same

Definition: A table using frequently asked questions with accessible responses for each option. To be included near the start, immediately after the decision is defined.

Rationale: Presenting the implications or consequences of each option in a parallel format rather than sequentially is in line with the IPDAS checklist to give a balanced presentation of information to avoid giving one option precedence (25). The template initially aimed to achieve this with a table to enable comparison of the “positive and negative features of the available options” (13). However, feedback from the patient panel indicated that framing the outcomes as positive or negative did not allow for individual differences in how people might perceive them, with some outcomes (such as still being able to get pregnant) possibly a benefit for some people but a disadvantage for others. Furthermore, feedback from an accessibility specialist (SS) suggested that the amount of information listed as benefits and disadvantages for each option made it difficult to compare the consequences. Instead, SS suggested using an a table format which presents the answers to frequently asked questions for each option, to enable easier, rapid comparison of related outcomes (26). This change was implemented, and the patient panel agreed it was both more accessible and more appropriate. Avoiding framing outcomes as either benefits or disadvantages and allowing people to decide for themselves presents an alternative way of implementing the IPDAS criterion of showing positive and negative features of each option (25).

The table was further simplified following feedback from the patient panel that where two options had the same response to a question, these boxes should be merged to save people reading the same information twice and trying to detect if there is a small difference or not. This is in line with guidance to reduce cognitive load as we only have limited capacity to process information about different options simultaneously (25), and it provides a novel approach to achieving this (See Box 2).

Box 2 **Example of the table showing implications of each option.**

**Table:** 

	Have the operation 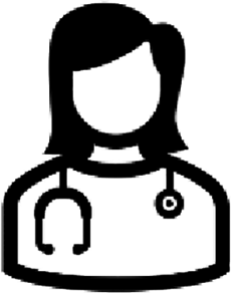	Do not have the operation 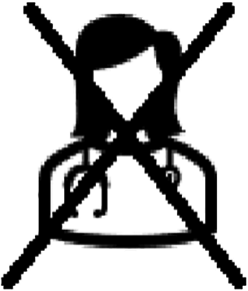	Wait to decide 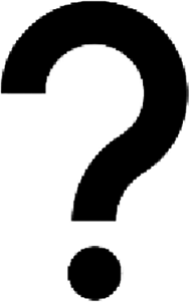
How would this affect my chance of developing cancer?	Would prevent womb, ovarian, fallopian tube and cervical cancer.	You would have a chance of developing womb, ovarian, fallopian tube or cervical cancer. The next page explains how your chance goes up as you get older.	
	You would no longer need the “smear test” for cervical screening.	Your chance is affected by a few things, e.g., it is higher if you are older, if you are overweight and if you are overdue for the “smear” test for cervical screening.

(3) Optional further details such as tailored risk, symptoms, and personal stories

Definition: The defining feature of this component is that it is optional, as some people may not want to read further information. The content of the further information may depend on the exact decision being made, but standard components included in this template are: tailored risk information, symptoms of the health condition, other people's stories, and specific further details relating to the decision in question such as possible side effects/outcomes (e.g., menopause) of taking a certain decision (See Box 3).

Box 3 **Example of presenting optional information More details.**

Some people like to know more about their options, to help make their choice.

You can click on any button below to find out more.

• What do the recommendations say?
• What are the chances of womb cancer and ovarian cancer?
• What are the symptoms of womb cancer and ovarian cancer?
• Information about the menopause
• Information about Hormone Replacement Therapy, known as HRT
• Other people's stories

Rationale: In early versions of the decision aid, users were tunnelled through information to support decision-making before they could access the table. However, the patient panel and public involvement consultations confirmed that people would like the essential information upfront as not everyone wants to read additional details. Therefore, the table was presented at the start of the decision aid, with the chance to read further supporting information afterwards if people were interested. This is in line with fuzzy-trace theory which suggests presenting key information first as people often make decisions based on a gist or feeling rather a rational consideration of all information (26, 27). It is also in line with IPDAS criterion to “highlight essential content, with the option for patients to explore more comprehensive information they find salient” (25).

Specific details about the lessons learned in how to present the optional components are included below:

Tailored risk:

• The literature recommends including a comparison group (e.g., general population) to help people interpret their risk statistics (28, 29), and the patient panel agreed this was important to facilitate understanding but encouraged presentation of this side-by-side with the at-risk population to enable easy comparison. This is consistent with evidence about effective risk communication (30)
• In line with recommendations for transparency, information was clearly provided about the time span over which a risk applied, e.g., life time vs. over the next ten years (28). The patient panel agreed this was important in order to ensure that people were not misled about the impact a decision could have.
• Whilst the evidence suggests framing risks both positively and negatively to avoid influencing people (29), our patient panel preferred less written information about risks. This is in line with another user-focused study which found that presenting risk information in multiple written formats could be confusing for people (31).

Personal stories: Personal stories have been defined as “stories, testimonials, or anecdotes that provide illustrative examples of the experiences of others that are relevant to the decision at hand” (32) and can be particularly liked by people with lower health literacy (33). Initial feedback from the public involvement consultations suggested that some contributors found personal stories engaging, therefore they were included in the decision aid template despite mixed evidence about their effectiveness (32).

The personal stories focused on the *process* of decision-making rather than the *outcomes* of decisions people had made, to minimise the risk of influencing people's choices (32, 34). The patient panel reported liking these personal stories, and their comments indicated that the stories may reassure people as well as increase engagement, suggesting that just seeing that someone else has been through the process of making the same decision can be comforting for people. We recommended including one story to describe each available option for a decision, to ensure the options were represented equitably in line with IPDAS (13).

(4) Values clarification activity, including tailored feedback

Definition: Items co-developed with target population to explore relevant personal values, with an algorithm to calculate personal feedback.

Rationale: The inclusion of values clarification methods to encourage decision-making in line with personal values is well established (20, 35), but this development process showed the importance of the exact wording of the values themselves. Patient panel and public involvement contributor feedback on values clarification activities adapted from existing decision aids indicated that the items could be seen as inappropriate or insensitive, which reduced their perceived value. For example, an item asking “I feel that taking action to lower my chance of getting bowel cancer is very important/slightly important/not important to me” was seen as inappropriate because it implies that someone at increased risk of cancer might not care about reducing their risk, and does not acknowledge that some people may not be *able* to take action due to other factors, such as age or co-morbidities. Therefore the identification of values to include in the values-based activity was directly informed by the issues raised when talking about the different options during our public involvement consultations, to ensure they were relevant to our specific population (36) (See Box 4).

Box 4 **Example of values clarification activity.**

**Table:** 

	Doesn't really matter to me	Matters to me a little	Matters to me a lot
Keeping my options open for becoming pregnant in future			

Feedback from our patient panel also revealed that a tailored summary about which option people were leaning towards was an expected output from this kind of “quiz”, and were disappointed when they were not rewarded in this way for doing the values clarification activity. Therefore, the decision aid template was adapted to use an algorithm to calculate to what extent someone is in favour of an option based on their values. This contrasts slightly with the preference for the table showing implications of each option to avoid imposing assumptions about what is a benefit for people, but is in line with evidence that people want to explicitly see how their values map on to the options available (20).

(5) Tailored summary to facilitate discussion with a healthcare professional

Definition: A tailored summary of patients’ preferences according to their values clarification activity, with explicit encouragement to bring a copy to discuss at the next appointment with a relevant healthcare professional. An open-text box for the user to add any questions for the healthcare professional, and links to relevant supporting material to help them consider what they might want to ask (See Box 5).

Box 5 **Example of an excerpt from the tailored summary to facilitate discussion with a healthcare professional**

Your answers suggest you are not sure if you want an operation to remove your womb, cervix, ovaries and fallopian tubes.

It's fine that you are not sure at the moment. These decisions are difficult and it's great that you’ve looked through the session today and are thinking about your options.

As you have some concerns about taking HRT, it will be important to talk to your gynaecologist and/or GP about this.

Rationale: The IPDAS recommend providing an output to facilitate shared decision making with a healthcare professional, such as a summary of users' preferences or values, key knowledge gained, or outstanding decisional needs e.g., by prompting the user to think about questions they might want to discuss with their healthcare professional (37). Interestingly, there is limited evidence for the effectiveness of this component in terms of improving informed decision-making, but this is due to a lack of research specifically addressing this question rather than refuting evidence (37). The Winton Centre's research in developing accessible genetic test reports similarly emphasises the importance of “actionability” or clear guidance as to what happens next (31).

The patient panel and public contributors agreed that it was very important that the decision aid was positioned as a tool to be used alongside conversations with a healthcare professional, rather than to reach a decision entirely independently. This was for safety reasons to ensure that all health-related decisions are made in consultation with appropriate specialists as part of shared decision-making. The tailored summary was seen as an important component to reinforce this by explicitly encouraging users to discuss their summary with a healthcare professional. The panel and stakeholders also suggested the type of healthcare professional referred to by the decision aid needs to be appropriate for discussing the decision in question, in order to avoid frustrating experiences of being referred around the healthcare system.

Table 4 provides an excerpt from the table of changes which shows how input from the patient panel, public contributors and stakeholders informed the development of the template, as well as some examples of more specific optimisations to the content. Decisions about the template were largely guided by the patient panel, public contributors and stakeholders specialising in accessible decision aids, whilst clinical stakeholders provided guidance on transparent, accurate information.

**Table 4:** Excerpt from table of changes.

Who	Informed decision aid template or specific content change	Negative comments	Neutral comments	Positive comments	Possible change	MoSCoW Must have Should have Could have Would like
Patient panel	Template: Define the decision	Needs something to put this session into context, to explain that there is a decision to be made and that this session can help you, rather than just starting straightaway with information about risk-reducing surgery.			Add a clear definition of the decision and the purpose of the session at the outset.	Must
Stakeholder	Template: table of implications of each option	The content of this table is good. There is however quite a lot of text/information in the 6 boxes. I wonder if you could present more like a table perhaps–where you have frequently asked questions in the left-hand column.			Change format of information about the different options to a table, to improve accessibility.	Must
Patient panel	Template: table of implications of each option	Still being able to become pregnant is not necessarily a benefit for everyone.			A table allows people to decide for themselves if something is a benefit or disadvantage for them.	Must
Patient panel	Template: table of implications of each option	The less text you have the better–suggest keeping the separate headings but put the text centrally underneath both where there is no difference, e.g., for don’t have the operation and wait to decide.			Combine the columns into one where there is no difference, to reduce the amount of information and avoid people reading the same information twice trying to spot what is different.	Should
Public contributor discussion group	Template: Optional further details	Some people want information about symptoms, some want information about HRT, some want patient stories–importance of optional information.			After presenting the table, allow people to read further about any particular topics that interest them.	Must
Public contributor discussion group	Template: Values clarification activity	The wording of the values clarification items is very important, some people felt examples from existing decision aids were not appropriate, e.g., “reducing my risk is important to me” grates on me. It's clunky. It's not just about it being important to me. The reasons for someone not to take aspirin will make the decision for them (e.g., age, co-morbidities). “Don't feel the need” implies you don't care.			Develop values clarification items with people from the target population, to ensure they are relevant and appropriate.	Must
Patient panel	Template: Values clarification activity	[Re values clarification activity] It leads you to expect you will receive tailored feedback from an algorithm based on your answers, like you do in magazines. You need to have something about why you’ve filled this in, or there's no point. People will expect it to tell them something.			Provide tailored feedback based on responses to the values clarification activity.	Must
Patient panel, public involvement contributors and stakeholders	Template: Tailored summary to facilitate discussion with a healthcare professional and overall focus on reducing negative emotions	Some people may be distressed when reading it, especially if relatively newly diagnosed. Need to be able to signpost them to support and encourage them to discuss decisions with a healthcare professional for safety reasons.			Positive, reassuring language throughout decision aid, and encourage people to discuss their decision with a clinician. Include a feedback sheet to take to the clinician with options to add questions.	Must
Stakeholder	Content	We remove the cervix too. This needs to be mentioned and discussed.			Clarified that the risk-reducing surgery also involves removing the cervix and included a diagram to show where it is.	Must
Stakeholder	Content	Risk of cancer if you don’t have operation would also be higher if you are overdue cervical screening.			Added this information to the table showing implications of each option.	Must
Patient panel	Content	Generally people prefer the word “chance” to “risk” as it doesn’t sound as scary.			Changed “risk” to “chance” throughout.	Should
Public contributors	Content	I want to know about the symptoms I need to look out for.			Included optional section about symptoms of the relevant cancer.	Should
Patient panel	Content	Could there be an icon for each of the 3 options, to make it easier to interpret?			Added icons to the columns of the table to help illustrate what each option means.	Should
Patient panel	Content	What does “normal activity” mean - not sure any woman could return to “normal” that quickly. NHS website says 6–8 weeks to fully recover.			Removed reference to “normal activity” and used information from the NHS website about recovery times.	Must

### Guiding principles

Discussions with our patient panel and the public involvement consultations informed the co-development of guiding principles, see Table 5. These guiding principles complemented the specific structure outlined above by defining how decision aids for this population can best be designed to promote engagement. Key design objectives included promoting trust, reducing distress, being comprehensive, personally relevant and accessible.

**Table 5:** Guiding principles for engaging target population in decision aid, co-developed with patient panel.

Intervention objective:	
The aim of the decision aid template is to support people with a genetic predisposition to cancer to make informed, values-based decisions about managing their risk of cancer.	
**User characteristics:**	
• Wide range of users in terms of specific risks, cancer history, cancer experiences within the family, and length of time since diagnosis of genetic predisposition.	
• Some people have lower health literacy and lower motivation to engage with risk management.	
• People have experiences of their GP not knowing about management options for their genetic predisposition, limited support beyond first diagnosis, and confusion due to inconsistent information.	
• Some people are quite anxious and don’t want to be frightened.	
**Design objectives to promote engagement**	**Key (distinctive) intervention features**
*Each objective should be targeted toward a particular behavioural issue*	*Features that will achieve the design objective, preferably features that make the intervention unique*
Promote trust in the decision aid as a credible source of information	Seek endorsement from charities
	Emphasise that the decision aid was developed by people with genetic predispositions
	Help people feel reassured that this is clinically safe advice, use existing information from credible health services where appropriate.
	Transparency about what is not known, to increase trust. Explain where evidence is limited or may change.
	Include citations for evidence, in line with IPDAS and to show people the intervention is evidence-based.
Support people to feel positive about their risk management decisions and reduce anxiety and distress.	Explain at the outset that it is a personal decision with no right or wrong.
	Use of positive language that emphasises the benefits of knowing your risk and the effectiveness of risk management options
	Stories of people who used their values to help inform their decisions
	Do not expose people to frightening information unnecessarily, e.g.,
	• This gene alteration has the highest risk of bowel cancer.
	• Public involvement contributors prefer the term “chance” to “risk”
	Signpost to support and encourage people to seek professional support that they are entitled to, e.g., gynaecologist appt
	Position the decision aid from the start as something you might want to talk about with your healthcare professional.
Include broad coverage of all topics that people may be uncertain about, even where information is regionally different or not currently clear.	Clear information about all aspects of risk management for genetic predisposition, including those that are not recommended by local guidelines but that people may have read about elsewhere.
	Show options in table form with clear information about possible outcomes
	Use a consistent format across each decision for clarity
Ensure intervention feels personally relevant	Make it clear on the very first page who the decision aid is for and what it will do
	Allow users to choose which information they are interested in
	Tailor risk information to user's characteristics
	Include stories from a wide range of people
	Involve a wide range of public involvement contributors and participants in intervention development
	Carefully develop content to ensure it is as personally relevant as possible, working closely with public involvement contributors
Accessible for everyone, including those with low health literacy	Risk presented in user-friendly ways, informed by evidence and public involvement
	Present information using fuzzy trace theory to show the gist first, and more information if desired.
	Definitions of medical words if hover over
	Use short sentences and active voice
	Low reading age when run through readability checker
	Put table showing implications of each option at the start to ensure people can access this information without having to read through everything first.

### Programme theory

**Figure 1:**
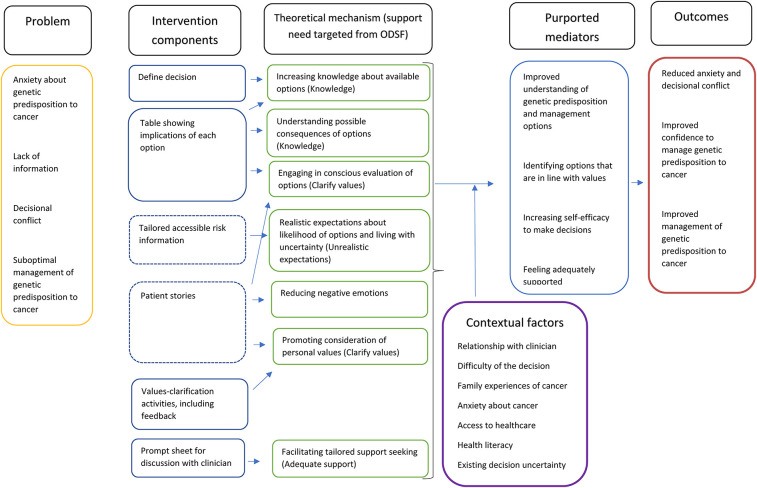
Programme theory for decision aid template.

The programme theory to show the mechanisms through which the decision aid template would support decision-making was developed bottom-up, informed by our discussions with the patient panel and public involvement consultations as well as evidence around shared decision-making processes (38). The mechanisms were subsequently mapped onto theory using the model of decision-making outlined by the Ottawa Decision Support Framework which draws on a range of decision-making theories and outlines four modifiable support needs that can reduce decisional conflict: knowledge, realistic expectations, clear values and adequate support (14), see Figure 1. While most of the decision aid mechanisms mapped on to the modifiable factors outlined by the Ottawa Decision Support Framework, negative emotions (such as distress) are described by the framework as a symptom of decisional conflict but not something that can be modified. However, this programme theory included managing negative emotions as a key modifiable mechanism.

In Figure 1, solid lines around intervention components indicate these are core components of the decision aid template, while dotted lines indicate they are optional components which users can choose to view if they are interested.

### Adaptation of the template for taking aspirin

The iterative process of adapting the template for another decisional context supported that the five components remained appropriate and relevant, the content could be easily adapted, and no new components were needed. However, it was still essential to work iteratively with relevant clinical specialists and our patient panel to co-design the specific decision aid content, with important optimisations including ensuring the items for the values clarification activity were salient and useful, and determining which optional further details people might want, such as clear communication about the current evidence for the benefit of taking aspirin.

## Discussion

This paper presents the first co-developed decision aid template for people to use independently to promote values-based decision making. The template has been named “PredispDA™” (Predisposition Decision Aid template, ©University of Southampton), and can be used to facilitate rapid development of decision aids for different predispositions. Our process shows how the selection and ordering of relevant components and specific content was driven by the user population and subsequently mapped onto theory and evidence rather than being deductively driven. This enabled important adjustments to the template to promote its acceptability to the target population, whilst still meeting all criteria from the IPDAS. Similarly, despite including comparable components to the Ottawa decision aid template, this template uses language and ordering which were user-led to increase acceptability to the target population.

### Building on IPDAS

Whilst this decision aid met all the IPDAS criteria, it was not guided solely by the IPDAS in terms of content. For example, a large section of the IPDAS focus on presenting probabilities, but this is only an optional component of the decision aid template for those who want to see it. Meanwhile the format and positioning of the table showing implications of each option to clearly present the outcomes of different options was a critical component of the decision aid template development which underwent several iterations based on feedback from the target population and stakeholders, but the IPDAS are quite open about how this is achieved only stating “The decision support technology makes it possible to compare the positive and negative features of the available options and shows the negative and positive features of options with equal detail (for example using similar fonts, order, and display of statistical information)” (13). The guidance provided in this paper about how to develop and present the core components of the decision aid template helps expand on the IPDAS by taking the target population's preferences into account.

### Personal stories

Personal stories are not included in the IPDAS due to the lack of clear evidence for any benefit to decision-making outcomes (32) and are not part of the Ottawa decision aid template, but they were liked by people during initial discussions and perceived to be reassuring and engaging. Therefore, narratives focusing on the decision-making process as opposed to the outcomes were an optional component of this template for users to read if they are interested. Guided by the patient panel, images were chosen to represent the person narrating each story to make the story seem more realistic, which influences the impact of the narrative (39). However, there is a risk that whichever narrative the user feels the strongest connection with may be more likely to influence their decision-making process, and images might exacerbate this situation by triggering feelings of similarity or difference in terms of narrators’ ethnic, gender or cultural identity (34). Real-time interviews with a wider group of people about their perspectives of these personal stories will be used to better understand how they could facilitate or hinder informed decision-making. If the images appear to influence people, more neutral illustrations created by an artist could be used which may not elicit such strong feelings of identity.

In addition to outcome and process narratives, a third type of narrative has been termed experience narratives, which are theorised to offer powerful insights to help people gain a more realistic understanding of what it would be like to follow a certain option, and possibly increase resilience (34). This could be an effective way to implement the IPDAS recommendation of helping people imagine the psychological, physical and social effects of each option (13), but, as with outcome narratives, could risk influencing people and introducing bias. This decision aid template avoided incorporating outcomes or experiences into the personal stories, and instead used them as an opportunity to model values-informed decision-making and reassure people. Considering the mechanism through which the stories were theorised to support people, alongside users' preferences, was useful for ensuring the content was consistent with its purpose.

### Values clarification methods

The mode of feedback from the values clarification activity was regarded as important to optimise its practical application to decision-making, with users expecting to see tailored feedback generated from their responses to the values items. Feedback on values clarification activities was not covered by the IPDAS, which only state that ‘The decision support technology asks patients to think about which positive and negative features of the options matter most to them (13), while the Ottawa Patient Decision Aid template suggests people select for themselves which option best aligns with their values (14). However, a recent review of values clarification methods does suggest that ‘multicriteria decision analysis’ be used in decision aids (20), which is defined as “The user is asked to directly indicate the extent to which a decision attribute or outcome matters to them or how good or bad they deem it to be. These values are then used in a model that calculates alignment between what matters to the user and the available decision options”. This suggests that both evidence and user preferences support the incorporation of personalised feedback on values clarification to enhance the usefulness of these methods in decision aids.

However, while attempts have been made to guide decision aid developers in which methods for clarifying values are most effective (20), less attention has been paid to the importance of the wording of the values themselves. In this case, detailed discussion with the target population was essential to ensure that items for values clarification were perceived as genuinely useful, relevant and as recognising the complexities of people's situations, while generically worded items such as “How important is it to you to reduce your risk of cancer?” (14) were seen as irrelevant and irritating which could reduce engagement with these activities.

### Strengths and limitations

The co-development process enabled detailed written and verbal input from a dedicated patient panel with an in-depth understanding of the decision aid's purpose and the development process. This was complemented by consultations with a wider group of public involvement contributors living with the genetic predisposition in question, which enhanced understanding of the specific needs and barriers to decision-making for the target population. However, self-reported demographics indicated that most of these public involvement contributors were white and aged between 30 and 60 years. Further work is needed to ensure the decision aid template is appropriate and supportive for people from different ethnic groups and ages, and we will begin to address this by purposively seeking diversity during our think-aloud interviews in the next phase of development. Participants in these interviews will also be asked if they are happy to complete a self-reported health literacy questionnaire, to enable exploration of the extent to which people with lower health literacy have inputted to the development of the decision aid template. However, we recommend that further work with underserved groups including people under 30 and over 70 years, different ethnic communities, LGBTQ+communities, socio-economically disadvantaged people, and people with learning disabilities or physical disabilities will be important to explore how the inclusivity of the template components could be improved. In addition, while public involvement suggests the decision aid template is highly acceptable for the target population, further work is needed using validated psychometric scales to explore the impact of this decision aid template on outcomes.

### Conclusions

A decision aid template called PredispDA™ (Predisposition Decision Aid) has been co-developed with people from the target population to provide key components and supporting guidance for anyone wishing to produce decision aids for people living with genetic predispositions. The template was developed through a robust and rigorous process which incorporated best practice guidance alongside in-depth co-development activities with the target population to produce a relevant and accessible template. This template has already been successfully adapted for another decisional context with close input from public involvement contributors and clinical stakeholders, supporting its potential to be further adapted for other genetic predispositions. The guiding principles help to ensure that adaptations to the content will remain engaging and appropriate for the target population, whilst the programme theory provides transparency about how the decision aid is theorised to work which should help developers ensure adaptations are consistent with hypothesised underlying mechanisms.

Given the importance of the target population's input to the co-development of this template, we believe that any adaptations would need close collaboration with people with lived experience of the genetic predisposition in question. The ADAPT guidance for adapting interventions for different contexts emphasises the importance of involving relevant stakeholders, and could be used to help guide this process (40).

While this template has provided a guide to developing key components of a decision aid, a challenge remains in how to provide and fund sustainable open-source software which can be readily updated to facilitate rapid development of digital decision aids for different conditions (41).

## Data Availability

The original contributions presented in the study are included in the article/**Supplementary Material**, further inquiries can be directed to the corresponding author/s.

## Patient Reference Panel

Caroline Dale, Sue Duncombe, Rochelle Gold, Sonia Patton, Warren Rook, Richard Stephens, Lesley Turner, Frankie Vale, Helen White, Ivan Woodward, Steve Worrall, Julie Young.

## International Lynch Decision Aid Stakeholders

Munaza Ahmed, Lyndsy Ambler, Antonis Antoniou, Stephanie Archer, Ruth Armstrong, Elizabeth Bancroft, Kristine Barlow-Stewart, Elizabeth Barnett, Marion Bartlett, Julian Barwell, Hilary Bekker, Dany Bell, Cheryl Berlin, Matilda Bradford, John Burn, Sarah Cable, Dharmisha Chauhan, Ruth Cleaver, Elizabeth Coad, Gayatri Connolly, Gillian Crawford, Emma Crosbie, Victoria Cuthill, Tabib Dahir, Karina Dahl Steffensen, Eleanor Davies, Glyn Elwyn, Mary Jane Esplen, D Gareth Evans, Pia Fabricius, Andrea Forman, Kaisa Fritzell, Claire Giffney, Joana Gomes, Rebecca Hall, Helen Hanson, Menna Hawkins, Deborah Holliday, Roberta Horgan, Karen Hurley, Margaret James, Ros Jewell, Sarah John, Siobhan John, Victoria Kiesel, Anna Koziel, Anjana Kulkarni, Fiona Lalloo, Helen Liggett, Aela Limbu, Kate Lippiett, Anne Lowry, Manami Matsukawa, Tracie Miles, Shakira Milton, Pål Møller, Kevin Monahan, Laura Monje-Garcia, Alex Murray, Jennie Murray, Kai-Ren Ong, Anbu Paramasivam, Alison Pope, Sarah Pugh, Gabriel Recchia, Nicola Reents, Peter Risby, Neil Ryan, Sibel Saya, Raza Sayyed, Salma Schickh, Lucy Side, Sian Smith, Tracy Smith, Dawn Stacey, Eriko Takamine, Katrina Tatton-Brown, Helle Vendel Petersen, Robert Volk, Jennifer Wiggins, Lisa Wilde, Jennet Williams, Catherine Willis, Elizabeth Winchester, Kristi Withington, Emma Woodward, Alice Youngs.
