# Supplementary material for: Person-based co-design of a decision aid template for people with a genetic predisposition to cancer
Source: Front Digit Health. 2022 Nov 23;4:1039701. doi: 10.3389/fdgth.2022.1039701 (PMC9743799; doi:10.3389/fdgth.2022.1039701)
Supplement: Supplementary file 1 [file Datasheet1.docx]

Supplementary material A. Chronological record of co-development procedures with patient panel, public involvement consultations and clinician/decision aid stakeholders.

| **Date** | **Who with** | **N of people involved** | **Format** | **Aims** | **Outcomes** |
| --- | --- | --- | --- | --- | --- |
| February 2021 | Patient panel | 8 | Online meeting | To review examples of existing decision aids and identify components that would be beneficial for the decision aid template. | Three decision aids were reviewed in small groups and features were identified which were liked or disliked. Discussions around how this would translate to the decision aid template helped inform design of initial decision aid template. |
| July-August 2021 | Patient panel | 4 | Online meeting | To plan wider public involvement activities to inform the development of the decision aid template. | Identified ideas for recruiting wider public involvement contributors and methods for co-facilitating the online consultations with our panel members. |
| August-September 2021 | Public involvement contributors | 19 (max 4 per meeting) | Online meetings and telephone calls | To discuss preferences for the decision aid structure and content with a wider group of people from the target population. | Explored perceptions and experiences of managing risk, and feedback on components from existing patient support resources. Identified aspects that were liked or disliked and the type of support people wanted. |
| December 2021 | Patient panel | 7 | Online meeting | To refine the structure of Version 1 of the decision aid template and to identify how best to engage people from the target population. | Discussed first version of risk-reducing surgery decision aid. All feedback was collated in a table of changes and used to improve the accessibility, relevance and usefulness. The panel also co-designed guiding principles with the researchers to inform how the decision aid would engage its target audience.  Following the meeting, the updated version of the decision aid was emailed to the panel to show how their feedback had been incorporated and invite further comments. |
| December 2021 | Patient panel | 4 | Email written feedback Version 1 | To incorporate specific content changes from the patient panel on Version 1. | Specific optimisations to Version 1 of the decision aid based on written feedback from panel members. |
| February 2022 | Patient panel | 2 | Email written feedback Version 2 | To incorporate specific content changes from the patient panel on Version 2. | Specific optimisations to Version 2 of the decision aid based on written feedback from panel members. |
| February 2022 | Stakeholders | 3 | Email written feedback v2 | To incorporate specific content changes from the stakeholders on Version 2. | Specific optimisations to Version 2 of the decision aid based on written feedback from clinical and decision aid specialists. |
| May 2022 | Patient panel | 7 | Online meeting | To continue to optimise the decision aid content. | The panel reviewed the third version of the risk-reducing surgery decision aid (now online) and provided detailed feedback about the website navigation, as well as the content itself. |
| May 2022 | Patient panel | 1 | Email written feedback Version 3 | To incorporate specific content changes from the patient panel on Version 3. | Specific optimisations to Version 3 of the decision aid based on written feedback from panel members. |
| July 2022 | Stakeholders | 4 | Email written feedback Version 3 | To incorporate specific content changes from the stakeholders on Version 3. | Specific optimisations to Version 3 of the decision aid based on written feedback from clinical and decision aid specialists. |
